# Congenital hyperinsulinism in infancy and childhood: challenges, unmet needs and the perspective of patients and families

**DOI:** 10.1186/s13023-022-02214-y

**Published:** 2022-02-19

**Authors:** Indraneel Banerjee, Julie Raskin, Jean-Baptiste Arnoux, Diva D. De Leon, Stuart A. Weinzimer, Mette Hammer, David M. Kendall, Paul S. Thornton

**Affiliations:** 1 Department of Paediatric Endocrinology, Royal Manchester Children’s Hospital, Oxford Road, Manchester, M13 9WL UK; 2 Congenital Hyperinsulinism International, Glen Ridge, NJ USA; 3 Reference Center for Inherited Metabolic Diseases, Necker-Enfants Malades Hospital, Assistance Publique Hôpitaux de Paris, Paris, France; 4 Division of Endocrinology and Diabetes, Department of Pediatrics, The Children’s Hospital of Philadelphia and the Perelman School of Medicine at the University of Pennsylvania, Philadelphia, PA USA; 5 Department of Pediatrics, Yale University School of Medicine, New Haven, CT USA; 6 Zealand Pharma A/S, Søborg, Denmark; 7 Congenital Hyperinsulinism Center, Cook Children’s Medical Center, Fort Worth, TX USA

**Keywords:** Congenital hyperinsulinism, Hypoglycemia, Caregiver burden, Challenges, Unmet needs

## Abstract

**Background:**

Congenital hyperinsulinism (CHI) is the most common cause of persistent hypoglycemia in infants and children, and carries a considerable risk of neurological damage and developmental delays if diagnosis and treatment are delayed. Despite rapid advances in diagnosis and management, long-term developmental outcomes have not significantly improved in the past years. CHI remains a disease that is associated with significant morbidity, and psychosocial and financial burden for affected families, especially concerning the need for constant blood glucose monitoring throughout patients’ lives.

**Results:**

In this review, we discuss the key clinical challenges and unmet needs, and present insights on patients’ and families’ perspective on their daily life with CHI. Prevention of neurocognitive impairment and successful management of patients with CHI largely depend on early diagnosis and effective treatment by a multidisciplinary team of specialists with experience in the disease.

**Conclusions:**

To ensure the best outcomes for patients and their families, improvements in effective screening and treatment, and accelerated referral to specialized centers need to be implemented. There is a need to develop a wider range of centers of excellence and networks of specialized care to optimize the best outcomes both for patients and for clinicians. Awareness of the presentation and the risks of CHI has to be raised across all professions involved in the care of newborns and infants. For many patients, the limited treatment options currently available are insufficient to manage the disease effectively, and they are associated with a range of adverse events. New therapies would benefit all patients, even those that are relatively stable on current treatments, by reducing the need for constant blood glucose monitoring and facilitating a personalized approach to treatment.

## Background

Congenital hyperinsulinism (CHI) encompasses a heterogeneous group of rare β-cell disorders, characterized by recurrent episodes of hyperinsulinemic hypoglycemia caused by dysregulated insulin secretion [1–4]. CHI is the most common cause of severe and persistent hypoglycemia in infancy and childhood, and is associated with an increased risk of seizures, developmental delay and permanent brain damage, with lifelong neurodisability if treatment is delayed [2, 3]. Thus, timely diagnosis and management of CHI are critical to minimize the risk of neurocognitive impairment [4]. The incidence of CHI is estimated to be approximately 1:28,000–1:50,000 in Western populations [1, 2, 5], but can be as high as 1:2,500 in populations with higher rates of consanguinity [1, 2].

Early signs of CHI are often subtle and non-specific, such as jitteriness, poor feeding, lethargy, a high-pitched or weak cry (based on the authors’ clinical experience). More serious signs reflecting neuroglycopenia include apnea, seizures, severe irritability, coma or status epilepticus [1, 6]. Hypoglycemia presents in the first week of life in the majority of cases (60–70%) and is revealed by seizures in about half of the patients [1, 2], 20–30% receive a diagnosis in the first year of life, and about 10% of patients present and receive a diagnosis after 1 year of age [1, 2] (and based on the authors’ clinical experience).

The etiology of CHI can be acquired or genetic. In the neonatal period, acquired forms are usually associated with conditions like perinatal stress or maternal gestational diabetes and are often transient [6, 7]. Genetic CHI may be due to single gene mutations in the insulin secretory pathway or in genes causing syndromes with multiple other associated factors (such as Beckwith–Wiedemann syndrome or Kabuki syndrome) [2]. Although transient/acquired forms usually resolve within days or weeks, they can still result in abnormal neurodevelopment in up to one-third of children [7, 8]. Reports about the incidence of pathogenic mutations in genotyped children with CHI vary widely, ranging from approximately 30% to 80% [2, 9–11].

Histologically, genetic CHI is classified as either focal, diffuse or atypical. The focal form of CHI is typically a small area of islet cell expansion with scattered and minimal exocrine tissues included, whereas the diffuse form involves the entire pancreas and is characterized by nucleomegaly of some islet cells [1, 2]. CHI is termed ‘atypical’ if the tissue histology is not characteristic of either of these forms, and can include the overgrowth pattern of Beckwith–Wiedemann syndrome and the islet cell nuclear enlargement patterns localized to specific regions [2, 12, 13].

Early diagnosis and effective treatment are pivotal for preventing neurocognitive impairment, which dramatically increases the cost of care and has the most significant impact on the lives of patients and their families [14]. While the neurodevelopment outcomes of focal versus diffuse forms of CHI are similar [7, 8], the focal form can be cured, whereas those with diffuse forms have ongoing risks of hypoglycemia [15]. This suggests that the initial hypoglycemia prior to treatment may be the most important time for brain damage. In addition to being very costly [14, 16], diagnostic delay also lowers the quality of life of patients and decreases the freedom of their families [14]. Medical therapy for the management of infants and children with CHI is limited [3]. Treatment options include one drug approved by the US Food and Drug Administration (FDA) and the European Medicines Agency (EMA), diazoxide, and off-label use of many other drugs, including somatostatin analogues, such as octreotide and lanreotide, sirolimus and glucagon [15, 17, 18] (shown in Table 1). A significant proportion of children with CHI do not respond sufficiently to the existing pharmacological therapies [3, 9, 19–21] and continue to experience hypoglycemia, or have a requirement for burdensome nutritional support such as continuous feeding through a gastric tube (data of the Hyperinsulinism [HI] Global Registry [22] and Table 3) to prevent hypoglycemia.

**Table 1:** Treatment options for CHI [15, 17, 18]

Type	Drug name	Mode of action
Diazoxide		Activates K_ATP_ channels of pancreatic β cells and maintains them in an open state, inhibiting insulin secretion
Somatostatin analogue	Octreotide LAR	Decreases secretion of insulin through hyperpolarization of β cells and inhibition of calcium channels
	Lanreotide	
Sirolimus (formerly rapamycin)		Inhibits the mTOR signaling pathway, potentially limiting the production of insulin from β cells
Glucagon		Promotes hepatic glucose production and increases blood glucose levels

CHI, congenital hyperinsulinism; K_ATP_, adenosine triphosphate-sensitive potassium; LAR, long-acting release; mTOR, mammalian target of rapamycin

CHI imposes a drastically different lifestyle on affected families, and is associated with significant morbidity, and psychosocial and financial burden. Thus, it has a fundamental impact on the everyday lives and well-being of patients and their families, especially in the early years after diagnosis [14, 22]. However, there is a lack of quantitative evidence on the quality of life of patients and their families, and CHI-associated costs in the peer-reviewed literature. The most significant challenges faced by families of children with CHI are around-the-clock care, the need for constant blood glucose monitoring, and access to effective treatment.

In this review, we describe the medical and non-medical challenges faced by clinicians and families affected by CHI and discuss the unmet needs in diagnosis and management of this rare disease.

## Main text

### Key challenges in CHI

#### Delayed diagnosis

Early identification of CHI and timely intervention are key to achieving glycemic stabilization from birth (Table 2). Despite advances in diagnosis and treatment of CHI, adverse neurodevelopmental outcomes in children with CHI have not significantly improved over the past decades [8]. The risk of brain damage is similar in children with transient forms of CHI and in those with permanent CHI. Therefore, neurological damage and developmental delays are not due to the type of CHI, but rather a consequence of the initial severe hypoglycemia. Consequently, there is an increased probability of neurocognitive impairment if prompt diagnosis and adequate treatment are delayed [8, 23]. A report published in 2020 has shown that the experience of the medical team may also be a contributing factor, because the risk of severe hypoglycemic brain injury is increased in babies born in a maternity hospital without a neonatal intensive care unit [24]. Deficits or delays in care by the primary care team, including delays in reporting abnormal clinical signs and delayed referral and medical review, probably play a part in neuroglycopenia and, while not explicitly stated in a majority of cases, are a likely contributor to litigation claims resulting from brain injury [25].

**Table 2:** Key challenges in CHI

Challenge	Impact	Possible solution
Early diagnosis and intervention	Delayed diagnosis leads to potential hypoglycemia-related neurological damage and developmental delays Neurocognitive impairment has a significant impact on the lives of patients and their families	Early screening for severe recurring and prolonged hypoglycemia from birth
Lack of awareness of signs associated with CHI in neonatal primary care providers	Signs and symptoms of CHI are missed or misinterpreted, leading to misdiagnosis or delayed diagnosis	Increasing awareness of the signs of CHI in all professions caring for neonates and infants
Limited availability of treatment options	Limited repertoire of treatment options for a very heterogeneous patient population, making medical management difficult for many patients	Development of new treatment options
Lack of responsiveness to available treatment options	For those patients who are unresponsive or only partially responsive to available treatment options, medical management is difficult Patients for whom medical therapy is failing require subtotal pancreatectomy, which is often associated with postsurgical diabetes mellitus	Development of new treatment options
Adverse events with available treatment options	Medical therapy is associated with concerning adverse events in some patients, including pulmonary hypertension with diazoxide therapy and necrotizing enterocolitis with somatostatin analogue therapy Other adverse events, such as hypertrichosis with diazoxide therapy, can be very distressing for patients and families, even though not of a life-limiting nature	Development of new treatment options
Glycemic monitoring	The continuous need for glycemic monitoring is very demanding on patients and families Measurements with blood glucose meters may not be performed frequently enough to detect all episodes of hypoglycemia The utility of CGM devices has not yet been established in CHI	Development of new treatment options that minimize the need for constant glycemic monitoring Rigorous evaluation of CGM meters in CHI

CGM, continuous glucose monitoring; CHI, congenital hyperinsulinism

Successful outcomes for patients depend on close coordination of care among pediatric endocrinologists, neonatologists, radiologists, pediatric surgeons, pediatric anesthesiologists, pathologists and other subspecialists who have significant experience with CHI [26]. Therefore, referral of children with suspected or confirmed CHI to specialized centers is of crucial importance for successful initial management and to improve long-term outcomes [26]. Centers of excellence offer patients and their families access to a multidisciplinary team with: the highest level of expertise; clinical experience with a large number of patients; a dedicated in-patient primary care team; surgical, imaging and genetic testing expertise; and many other benefits that are likely to have a direct impact on improved outcomes in patients and families’ experiences [27, 28]. In June 2021, CHI International announced the first group of centers designated ‘Centers of Excellence’ for providing the highest quality of care for patients with CHI and their families around the world [28, 29]. However, despite their importance in providing expert care for patients with CHI, few of these specialized centers exist. There is a need to develop a wider range of centers of excellence and networks of specialized care to optimize the best outcomes both for patients and for clinicians. In addition, health insurance providers must be willing to authorize patients’ referral to centers of excellence without delay. For children with rare diseases, health insurance status has a notable impact on the continuum of care, and delays or denials of insurance companies have the potential to negatively impact children’s health [30].

**Fig. 1:**
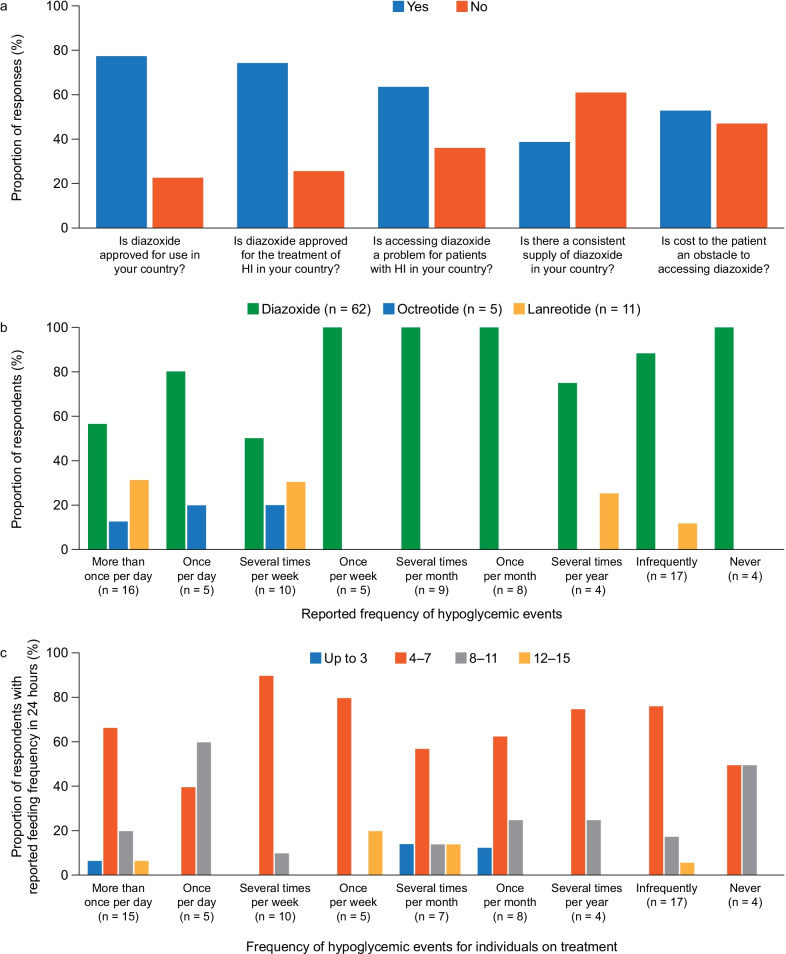
Data from the HI Global Registry global access survey, ‘Medication Management’ survey, ‘Glucose Monitoring’ survey, ‘Diet and Feeding Management’ Survey [45]. **a** Responses of 83 pediatric endocrinologists in 45 countries globally to questions about access to diazoxide in their country (Algeria, Argentina (2), Australia (2), Bangladesh, Belgium, Brazil (2), Bulgaria (3), Canada (5), Chile, China, Colombia (4), Egypt, Georgia, Germany (3), Ghana, Greece, Haiti, Hong Kong, Hungary, Iceland, India (9), Iraq (2), Israel, Japan, Kosovo, Lebanon, Luxembourg, Mexico (2), Montenegro, Myanmar, the Netherlands, Peru (6), Romania, Russia, Saudi Arabia, Serbia (2), Spain, Sudan, Sweden, Switzerland, Tanzania, United Kingdom (3), Ukraine (2), USA (4), Venezuela). **b** Reported frequency of hypoglycemia (plasma glucose < 4.0 mmol/L) and current medication use (n = 78). **c** Reported feeding frequency in 24 h and frequency of hypoglycemia (plasma glucose < 4.0 mmol/L) for individuals on CHI treatment (n = 75). HI, hyperinsulinism

Access to medical therapy is often inconsistent, even in developed countries. In a global access survey carried out by CHI International starting in November 2018 among 83 pediatric endocrinologists from 45 countries, the respondents indicated that, on average, 62% of their patients with CHI required diazoxide. Overall, 64% of the clinicians stated that their patients had problems accessing diazoxide in their country, despite 74% responding that diazoxide was approved for CHI in their country (Fig. 1a). Specific barriers to access were having no consistent supply of diazoxide in their country (response given by 61% of respondents) and cost to the patient (response given by 53% of respondents) (Fig. 1a) [31].

For many patients with CHI diagnosis is not rapid [14], and families may find it difficult to gain access to doctors who have experience with CHI and a sufficient understanding of the disease to provide the best care. Despite existing recommendations for diagnosis and treatment [32], there are frequent delays in recognizing the often subtle signs of hypoglycemia, leading to potentially avoidable neurological damage. In addition, guidelines for endocrinologists and neonatologists often provide conflicting recommendations, introducing additional uncertainty leading to delays in diagnosis. It is unsurprising that a national rare disease framework published in 2021 identified delayed diagnosis as a top priority for various stakeholders, including patient groups [33]. There is a clear need to improve recognition of pathological hypoglycemia from the normal transitional hypoglycemia in the first few hours of life, to initiate prompt and effective treatment to prevent the risk of neuroglycemia-induced brain injury [23].

In the authors’ opinion, a better screening system for neonates is needed to identify severe and recurrent hypoglycemia from birth, and to initiate treatment to normalize glycemia as quickly as possible. There is also an urgent need to spread knowledge among neonatal primary care providers to enable prompt identification of the more subtle signs of CHI, initiate rapid intervention, and enlist support from endocrinologists before irreversible brain injury occurs. Considerable controversy has been introduced by clinical practice guidelines and published reports that have proposed diagnostics pathways and parameters, that may in fact prevent prompt recognition of recurring and prolonged hypoglycemia and delay diagnosis of CHI [34, 35]. These are often weighted in favor of minimizing the number of investigations for common transitional forms of neonatal hypoglycemia, with less emphasis on diagnostic ascertainment of CHI [34]. In some settings, rapid turnaround genetic testing (4–7 days) may help to quickly determine if an individual patient requires immediate referral to a specialized center, and can play an important role in the clinical management of these children [9]. Furthermore, a consensus on treatment guidelines would help to inform decision-making and align treatment choices for patients.

#### Limited efficacy of available treatment options

Therapeutic options for CHI include both medical therapy and surgical intervention; however, only a limited number of drugs are approved for the treatment of CHI and several pharmacological options are used off-label [36] (Table 1). Many of these treatment options are not available globally, owing to a lack of approval by local regulatory authorities or other access barriers [36]. The first-line therapy for CHI is the adenosine triphosphate-sensitive potassium (K_ATP_) channel activator diazoxide, the only therapy for CHI approved by the FDA and the EMA [37, 38]. Diazoxide was also included in the Model List of Essential Medicines for Children in 2019 of the World Health Organization (WHO) for hypoglycemia [20, 39, 40]. The therapeutic dose range of diazoxide is relatively wide and can be adapted to the severity of disease [18]. However, only 41–64% of children with persistent CHI have been shown to be responsive to diazoxide therapy [10, 41, 42], whereas a higher proportion of cohorts that include children with acquired forms of HI respond to diazoxide [43, 44]. Up to 82% of children with CHI who are unresponsive to diazoxide have inactivating mutations of the genes encoding K_ATP_ channels (K_ATP_-HI) [4, 19], the most common and severe forms of genetic CHI [3, 4, 9, 21]. Approximately 70% of children with homozygous or compound heterozygous recessive K_ATP_ mutations are unresponsive to medical therapy and require pancreatectomy [9]. In up to 40–60% of children with K_ATP_-HI, the defect is limited to a focal lesion that can be cured with localized surgical excision [18, 21].

Among those children who receive diazoxide therapy, many continue to experience frequent hypoglycemic events, necessitating frequent feeds to maintain normoglycemia. There is no clear consensus on the frequency of hypoglycemia that is acceptable and incurs the least risk of brain injury. Data on the frequency of hypoglycemic episodes from the HI Global Registry collected between October 2018 and April 2021 show that approximately 27% (21/78) of patients with CHI on therapy had hypoglycemic events (plasma glucose < 4.0 mmol/L) at least once a day, and an additional 19% (15/78) experienced hypoglycemia at least once a week (Fig. 1b) [45]. These data are based on patient reports and, because blood glucose testing is infrequent in some patients, these patient reports may underestimate the true frequency of hypoglycemia. Among patients on treatment who had hypoglycemic events once or more than once a day (n = 20), overall 60% (12/20) had 4–7 feeds, 30% (6/20) had 8–11 feeds and 5% (1/20) even had 12–15 feeds in 24 h (Fig. 1c) [45].

For those children who are unresponsive to diazoxide, treatment options are limited, and clinicians are often obliged to innovate and repurpose other classes of medications, many of which are not specifically approved for use in CHI. Second-line medical therapy includes the use of somatostatin analogues, usually octreotide [46], although this class of therapy is not approved for use in CHI. In children with diffuse disease in whom medical therapy has failed, subtotal pancreatic surgery may be required, which is associated with postsurgical complications such as development of insulin-dependent diabetes mellitus [4, 18]. In adolescence and adulthood, a large proportion (up to 91%) of patients who underwent > 95% pancreatectomy in infancy will develop diabetes [47, 48].

#### Adverse events of treatments

Owing to the very limited treatment options for CHI, off-label use of medications is common. However, many of these therapies have not been formally tested in patients with CHI in well-controlled clinical trials that might better inform benefit–risk decisions (Table 2). As a result, many patients can be affected by limited or uncertain efficacy and clinically concerning adverse events, such as those that have been observed with sirolimus [17, 49]. Even long-established treatments may carry certain risks, such as necrolytic migratory erythema and canula obstruction observed with glucagon therapy [50].

In the acute treatment phase, diazoxide therapy causes fluid accumulation and may be complicated by pulmonary hypertension, neutropenia, thrombocytopenia and pericardial effusion; although the latter are uncommon [2]. The most frequent side effect associated with prolonged diazoxide treatment is hypertrichosis; additional adverse events include nausea, anorexia and bad taste in the mouth [15, 23, 51]. Although not considered to be of a life-limiting nature, the excessive growth of body hair can be extremely distressing for patients, especially for older children, and may lead to stigmatization and subsequent discontinuation of diazoxide treatment [2, 15]. Diazoxide also causes coarsening of facial features, with some families reporting that children on long-term treatment develop similar facial features to one another [2].

Octreotide therapy needs to be monitored carefully, particularly at therapy initiation, for deterioration of liver function [2]. Acute side effects include diarrhea, risk of necrotizing enterocolitis in newborns due to its effect on splenic circulation, and acute hepatitis [2, 15, 52, 53]. More long-term side effects include suppression of thyroid-stimulating hormone and growth hormone, and the development of gall bladder sludge or stones [2, 15, 26]. In some children, mild growth deceleration was observed, which resolved in most children after treatment discontinuation [2, 15, 26].

#### Glycemic monitoring

Maintaining normal blood glucose levels is the key treatment goal in CHI, but glycemic monitoring remains a significant challenge for families (Table 2). Monitoring plasma glucose by fingerprick with handheld glucose meters can be conveniently done at home; however, measurements may not be performed frequently enough to detect all episodes of hypoglycemia [2, 26]. Currently, there is no specialized equipment available for reliable home-based blood glucose monitoring in the hypoglycemic range. Continuous glucose monitoring (CGM) devices provide an alternative approach for subcutaneous glycemic monitoring that can increase the frequency of monitoring and may be especially useful at night [2, 54]. Although the accuracy of CGM devices continues to improve, they are known to be less accurate in the hypoglycemic range and are liable to pressure-induced sensor attenuations in this range, potentially leading to false low-sensor readings [55–58]. Overall, their reliability in the hypoglycemic range has not been rigorously evaluated in the CHI setting [2, 54].

The key challenges in CHI are summarized in Table 2.

### The impact of nutritional support requirements on caregiver and disease burden

Patients with CHI commonly require dietary carbohydrate supplementation in addition to medical therapy to avoid hypoglycemia [51]. Initial glucose support is typically provided by intravenous infusions, which necessitate a central venous catheter for delivery of high concentrations of glucose (12.5–30%, based on the authors’ clinical experience), requiring patients to remain in hospital and causing an increased risk of infection and thrombosis from the catheter [3]. Increased intravenous fluid volumes in newborns can result in fluid overload and potentially in cardiac failure [3], which can be exacerbated by diazoxide therapy [18]. After the initial stabilization period, the carbohydrate supplementation may be provided by regular glucose-enriched oral feeds or continuous enteral feeds via percutaneous gastrostomy or nasogastric tubes. Patients who rely on continuous enteral feeding through a gastric tube to maintain normoglycemia may experience severe hypoglycemia if interruption of continuous feeding were to occur for any reason.

Multifactorial feeding aversions affect a large proportion of children with CHI [2]. Data from the HI Global Registry 2021 annual report show that 69% of 132 families contributing to the Diet and Feeding Management Survey reported one or more feeding issues [59]. The feeding problems most frequently reported were poor appetite (40%) or refusal to eat (39%), while gastroesophageal reflux, gagging, problems with texture, vomiting and slow eating were each reported by over 26% of families [59]. These feeding difficulties pose a significant burden for families [2, 51], requiring careful meal planning, which may cause issues with meal delivery by other caregivers such as school or daycare. Parents’ stress and anxiety about feeding often aggravate the child’s feeding aversion. This may lead to a disturbed relationship with food, with many patients eating only a limited food selection even as adults [60] (and based on CHI International’s experience with the CHI patient community).

The patient’s dependence on nutritional support affects the ability of them and their families to lead normal lives, and limits the child’s participation in normal social interactions such as school, birthday parties, play dates and sleepovers. Activities need to be scheduled around meals or feeds, and must be frequently interrupted to check blood glucose levels.

Babies or children dependent on continuous feeds through gastrostomy tubes are inhibited in their ability to play and develop normal motor skills, and children connected to infusion pumps with an accompanying large backpack have a reduced ability to move and travel normally (based on CHI International’s experience with the CHI patient community).

Parental fear of hypoglycemia and the associated risk of neurological damage causes stress for the family and often leads to eating disorders in the children or overfeeding, which, together with limited physical activity, may result in obesity. Many children become overweight in early life, although this may improve over time (based on CHI International’s experience with the CHI patient community).

The nutritional support-related factors that have an impact on caregiver and disease burden are summarized in Table 3.

**Table 3:** Factors related to nutritional support that have an impact on disease burden

Frequent feeds or meals with specific nutritional requirements
Feeding difficulties
Risks and discomforts associated with invasive delivery methods (intravenous glucose, nasogastric tube, gastrostomy)
Parental anxiety over sufficient feeding to avoid hypoglycemia
Negative impact on normal daily activities and social interactions

### The psychosocial burden of CHI

**Fig. 2:**
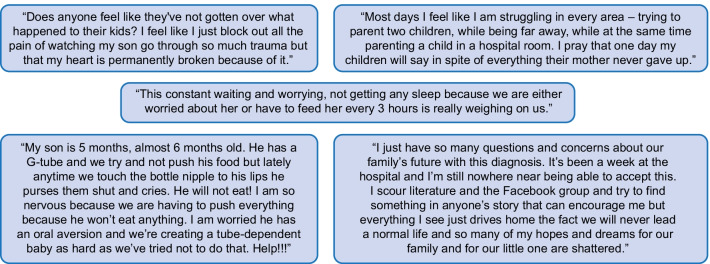
Deidentified quotes of parents of children diagnosed with CHI (provided by CHI International). CHI, congenital hyperinsulinism

CHI causes a significant psychosocial burden for patients and their families and caregivers, yet the peer-reviewed literature largely lacks quantitative assessments of the emotional and social cost associated with CHI. An excerpt of quotes of parents of children with CHI is presented in Fig. 2, demonstrating the significant impact a CHI diagnosis can have on families.

Constant vigilance is required to avoid hypoglycemia, which imposes a drastically different lifestyle on families and extracts a constant emotional cost of care [14]. The situation is similar for children with diffuse disease who develop lifelong diabetes as a result of subtotal pancreatectomy [14]. For some people in the CHI community, it has been reported to CHI International that the fear of hypoglycemia never goes away and is a constant worry, even when post-pancreatectomy patients transition to diabetes. The hypervigilance from the pre-diabetes phase of CHI often continues. More research is needed into this subject. Complex medical therapy and feeding regimens and the continuous need for blood glucose monitoring, no matter the time or place, are taxing for families and adversely impact on a child’s normal social interactions (based on CHI International’s experience with the CHI patient community).

The HI Global Registry 2021 annual report data demonstrate that parents of children with CHI who completed the Parent Quality of Life Survey reported that the management of CHI can be’demanding’ in all age groups. This was most notable in the under 5 years age group (49% [25/51]), but was similarly evident for the 5–11 years age group (38% [18/47]) and in the 12 years and older age group (39% [9/23]) [59]. Notably, at least 19% of parents in each age group described the daily management of CHI as ‘complicated’ [59].

The unpredictability of the disease, persistent fear of hypoglycemia, and the worry about neurological damage and developmental delay are particularly burdensome for families. Regardless of the child’s age, most parents (82% [100/122]) reported that they worry about their children [59]. The fear and uncertainty in the initial period of diagnosis are especially demanding. Prolonged hospitalizations until an effective treatment is identified can require parents to be away from home and work for long periods of time [14]. Most long-term follow-up studies have focused on neurodevelopmental outcomes and time to resolution, but there are few descriptions of the natural history of CHI with regard to growth, weight, feeding or medication use [61].

Owing to CHI-associated developmental delays, children with CHI may require additional support at school. Even in stable patients with relatively successful treatment, there is a constant need for blood glucose monitoring, requiring teachers to receive specialized training, which is not always available in a timely manner [14]. As a result, parents often need to spend considerable time at school until teachers are proficient in providing support. If teachers are not trained, or if training is delayed, children are often sent home at the first sign of feeling unwell, which leads to lost schooling as well as additional stress for the family [14].

It is well recognized that many children with CHI have minor developmental needs, for example in domains of self-help [62], which may not be identified unless standard batteries are used to investigate cognitive and behavioral components of neurodevelopment. Such children may also require additional support, for a variety of reasons, for different aspects of their education and learning.

Other people’s unfavorable reactions to the visible signs of the disease, such as the backpack for the infusion pump, nasogastric tubes or excessive body hair, represent an additional burden for families and contribute to an already demanding way of life. Those patients who suffer from postsurgical exocrine pancreatic insufficiency require enzyme replacement therapy and often exhibit symptoms of malabsorption, which can contribute to increasing emotional problems as they grow older. Furthermore, the presence of gastrostomy/nasogastric tubes or obesity from frequent feeding may negatively impact the child’s body image, especially in older children (based on CHI International’s experience with the CHI patient community).

### Caregiver burden

The medical and nutritional management and the constant vigilance required to care for children with CHI are extremely demanding on caregivers, with the burden of care often disproportionately affecting mothers (based on CHI International’s experience with the CHI patient community). Parents caring for children with CHI report about constant anxiety associated with ensuring that their children feed enough to avoid neurocognitive impairment when faced with feeding aversions and other feeding difficulties, and the negative impact of lack of sleep due to nightly glucose monitoring (based on CHI International’s experience with the CHI patient community).

The HI Global Registry 2021 annual report provides survey data on how many parents of children with CHI felt that their lives were’ruled by HI’. Overall, 70% (36/51) of respondents with children below 5 years of age feel that their lives are ‘quite often’, ‘very often’ or ‘always’ dominated by HI. This proportion decreased to 58% (28/48) in parents of 5–11-year-old children and to 34% (8/23) for parents with children 12 years of age or older [59]. Furthermore, 48% (59/123) of parents of children across age groups reported that their physical health has suffered from having a child with CHI, whereas over two-thirds of parents (67% [82/123]) responded that their mental health has suffered [59] (individuals were included in this analysis if they answered that their physical or mental health suffered ‘somewhat’, ‘quite a lot’, or ‘very much’).

The data compiled from the respondents’ answers to the HI Global Registry 2021 annual report Parent Quality of Life Survey illustrate that having a child with CHI can also have a significant impact on family planning and relationships. Among 123 respondents, 36% chose not to have any additional children and 19% were delaying having additional children [59]. Of 112 parents in relationships who responded, 58% reported that having a child with CHI strengthened the relationship with their partner, but 17% reported a negative impact on their relationships and for 5% their relationship ended [59].

Despite this, parents of children with CHI rate their own quality of life as high. Among 123 parents who reported on their own quality of life, 78% (41/52) of those with children under 5 years of age rated their quality of life as ‘good’, ‘very good’ or ‘excellent’. This proportion increases to 83% (40/48) for parents of children 5–11 years of age and to 91% (21/23) for those with children 12 years of age or older [59]. Equally, a report on a population-based cohort of children with permanent and transient CHI found that living with CHI was not associated with reduced health-related quality of life in childhood and adolescence compared to reference values [63].

### Financial burden

CHI is associated with substantial financial burden due to the direct and indirect costs of medical therapy, nutritional support, hospitalizations, outpatient medical assistance, cognitive disabilities, lost productivity, and healthcare utilization due to caregivers’ mental health problems and associated difficulties [14, 16]. Despite this, further research is required in this area to fully elucidate the financial burden of CHI.

For families, there is a substantial cost of care at and shortly after the time of diagnosis, which sometimes requires extended hospital stays while an effective therapy is established. During this time, families may have significant out-of-pocket expenses for hospital visits and may experience loss of income owing to the prolonged absence from work [14]. Many patients experience developmental delays because of delayed diagnosis or comorbid conditions, requiring at least one parent to act as full-time caregiver. The around-the-clock care needed for severely affected patients limits the main caregiver’s ability to work, often requiring them to leave their job which aggravates the existing financial hardship [14]. In addition, many families pay for additional therapy for their children, e.g. physical therapy or occupational therapy, out of their own pocket [14]. Factors contributing to financial burden for families are shown in Table 4.

**Table 4:** Factors contributing to the financial burden for families

Out-of-pocket expenses during the initial phase of diagnosis and treatment stabilization
Cost of travel for frequent hospital visits
Loss of income for the main caregiver
Cost for additional therapies, such as physical therapy

Despite being a rare disease, CHI is very resource intensive for both families and healthcare systems, but the true economic burden is poorly understood. Few cost of illness (COI) studies are available to inform policy-makers on key decisions regarding the provision of services [16]. A pivotal study on the annual COI for patients with CHI receiving care through the UK’s National Health Service (NHS) from 2015 to 2017 found that the total annual COI was £3.4 million [16], or approximately US$5.5 million. About £2 million of this cost (59%) was attributed to patients with diffuse disease who required surgical management. Patients below 1 year of age also represented a disproportionate share of the total cost compared with other age groups. Patients under the age of 1 accounted for only 4.4% (95/2146) of all patients with CHI, yet they incurred 61.8% (approximately £2.1 million) of the total annual cost of CHI care to the NHS [16]. Furthermore, unresponsiveness to first-line therapy and development of insulin-dependent diabetes mellitus after surgery and associated healthcare costs were identified as additional major cost drivers [16]. While COI appears high, it must be recognized that calculations are an underestimate of the true COI of CHI. This is likely to be considerably higher if also recognizing the impact of neurologic sequelae of the disease over the lifespan of the patient.

## Conclusion

CHI is associated with significant psychosocial and financial burden for families, yet valid tools for the assessment and quantification of patient and family quality of life are currently lacking. Caring for a child with CHI is extremely demanding and requires constant vigilance to avoid potentially life-limiting neurological damage, which is often exacerbated by feeding difficulties. Many parents of children with CHI report about their constant worry and anxiety, negatively affecting their mental health. Parental behaviors and reactions can have a considerable impact on their children. It is unclear what potential effect parental stress and anxiety could have on behavioral outcomes in their children. Contributing to an already stressful way of life is the financial hardship imposed by the disease, especially during the early phase of diagnosis and treatment stabilization. The most important drivers for CHI-associated costs include the period of time in initial diagnostic evaluation, lack of response to first-line therapy, postsurgical diabetes mellitus and lifelong neurodisability [16].

Regardless of treatment, there is a significant burden associated with CHI for all patients and families, especially concerning the need for constant blood glucose monitoring throughout patients’ lives [14]. For a large proportion of patients, the current treatment options are insufficient to manage the disease effectively. Even those patients who are relatively stable on effective treatment are looking for new therapy that can minimize the large impact constant blood glucose monitoring has on their lives [14]. New treatment options are necessary to provide an individualized approach to the extremely heterogeneous CHI patient population. The patients who would benefit most from new therapeutic alternatives are patients not responding to currently available treatments, thus requiring frequent oral or enteral feeding, as well as newborns and young infants. With agreed treatment guidelines, non-responders could be treated more effectively, with surgery only having to be considered as a last option.

In conclusion, healthcare programs need to be revised to enable those affected by CHI to have the best possible quality of life, including efficient screening to reduce time to diagnosis, more effective emergency treatment at the time of diagnosis, more rapid transfer to centers of excellence, and increased awareness of CHI across all specialties involved with newborns and infants. Finally, access to medicines and treatment guidelines that could be followed all over the world are needed urgently.

## Data Availability

Data sharing is not applicable to this article. No data sets were generated or analyzed during the current study.

## Abbreviations

CGM: Continuous glucose monitoring
CHI: Congenital hyperinsulinism
COI: Cost of illness
EMA: European Medicines Agency
FDA: Food and Drug Administration
HI: Hyperinsulinism
K_ATP_: Adenosine triphosphate-sensitive potassium
NHS: National Health Service
WHO: World Health Organization
