# Correction to: Congenital hyperinsulinism in infancy and childhood: challenges, unmet needs and the perspective of patients and families

**DOI:** 10.1186/s13023-022-02363-0

**Published:** 2022-05-18

**Authors:** Indraneel Banerjee, Julie Raskin, Jean-Baptiste Arnoux, Diva D. De Leon, Stuart A. Weinzimer, Mette Hammer, David M. Kendall, Paul S. Thornton

**Affiliations:** 1 Department of Paediatric Endocrinology, Royal Manchester Children’s Hospital, Oxford Road, Manchester, M13 9WL UK; 2 Congenital Hyperinsulinism International, Glen Ridge, NJ USA; 3 Reference Center for Inherited Metabolic Diseases, Necker-Enfants Malades Hospital, Assistance Publique Hôpitaux de Paris, Paris, France; 4 Division of Endocrinology and Diabetes, Department of Pediatrics, The Children’s Hospital of Philadelphia and the Perelman School of Medicine at the University of Pennsylvania, Philadelphia, PA USA; 5 Department of Pediatrics, Yale University School of Medicine, New Haven, CT USA; 6 Zealand Pharma A/S, Søborg, Denmark; 7 Congenital Hyperinsulinism Center, Cook Children’s Medical Center, Fort Worth, TX USA

## Abstract

Congenital hyperinsulinism (CHI) is a rare disease that causes newborn babies and children to have low blood sugar because of the abnormal release of insulin. Insulin is a hormone produced by the pancreas that promotes the transfer of sugar from the blood into the body’s cells. In a healthy person, insulin is released only after a meal when the level of blood sugar is high, but infants and children with CHI make insulin even if the blood sugar is low. This can lead to dangerously low blood sugar levels, which can cause brain damage if left untreated. Unfortunately, diagnosis and treatment are often delayed, resulting in avoidable brain damage and developmental delays in these children. CHI is associated with substantial stress and anxiety for the families, especially due to the need for frequent feeding and the fear of low blood sugars added to the constant need to measure blood sugar levels.

This article discusses the most important challenges and unmet needs in this rare disease, including the limited treatment options, the side effects of available treatment options and the heavy psychological, social and financial burden on affected families. Effective screening of newborns for CHI needs to be improved, and quick referral to specialized treatment centers is necessary to ensure the best outcomes for patients and families. In addition, awareness of CHI has to be raised in all medical professions caring for newborns and infants, and new medications are urgently needed to ensure the best possible treatment for all patients with CHI.

## Correction to: Orphanet Journal of Rare Diseases (2022) 17:61 https://doi.org/10.1186/s13023-022-02214-y

Following the publication of the original article [1], we were informed that the Plain Language Summary had inadvertently been omitted during typesetting.

The Plain Language Summary is shown here below and has already been added back to the original article.
